# Transparent OLED displays for selective bidirectional viewing using ZnO/Yb:Ag cathode with highly smooth and low-barrier surface

**DOI:** 10.1038/s41377-024-01739-0

**Published:** 2025-01-24

**Authors:** Eun-young Choi, Sung-Cheon Kang, Kanghoon Kim, Su-Hyeon Lee, Jeong-Beom Kim, Jang-Kun Song

**Affiliations:** 1 Department of Electrical and Computer Engineering, Sungkyunkwan University, Suwon-si, Republic of Korea; 2 Department of Semiconductor and Display Engineering, Sungkyunkwan University, Suwon-si, Republic of Korea

**Keywords:** Organic LEDs, Displays

## Abstract

Transparent organic light-emitting diode (TrOLED) displays represent cutting-edge technology posed to significantly enhance user experience. This study addresses two pivotal challenges in TrOLED development. Firstly, we focus on the innovation of transparent cathodes, a fundamental component in TrOLEDs, by introducing a ZnO/Yb:Ag cathode. This cathode employs a combination of seed layer and metal doping techniques to achieve a highly uniform surface morphology and a low surface energy barrier. The optimized Yb:Ag cathode on ZnO, with a mere thickness of 15 nm, exhibits remarkable properties: an extremely low surface roughness of 0.52 nm, sheet resistance of 11.6 Ω ϒ^−1^, an optical transmittance of 86.7% at 510 nm, and tunable work function (here, optimized to be 3.86 eV), ensuring superior electron injection capability. Secondly, we propose a novel TrOLED pixel structure that features selective bidirectional viewing, allowing different types of information to be selectively displayed on each side while preserving overall transparency and minimizing pixel complexity. This design innovation distinguishes itself from conventional TrOLEDs that display images on only one side. The bidirectional TrOLED design not only enhances openness and esthetic appeal but also holds promise for diverse applications across various user environments.

## Abstract (web summary)

A novel transparent OLED pixel geometry featuring a ZnO/Yb:Ag cathode enables selective bidirectional viewing, presenting different types of information on each side while maintaining transparency and simplifying pixel complexity.

## Graphical abstract

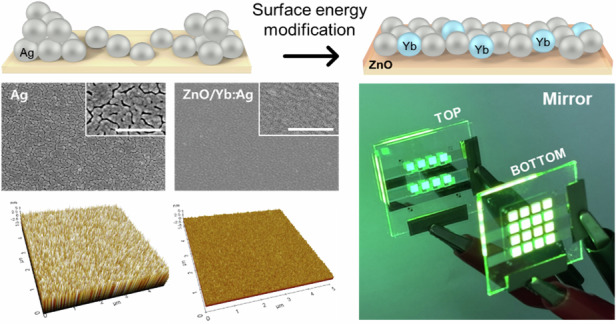

## Introduction

Conventional display devices, such as televisions and monitors, are opaque, obstructing the view of objects behind them and causing spatial discontinuity, thereby reducing openness. When turned off, these displays appear black, negatively affecting the aesthetic appeal of indoor spaces. In contrast, transparent display devices offer an enhanced user experience by improving spatial openness and allowing for harmonious integration with the surrounding furniture and objects. As a result, transparent displays can simultaneously provide information and maintain openness in various settings, such as train or car windows, electronic docents in museums and galleries, and showcases in stores^1,2^.

Organic light-emitting diodes (OLEDs) offer a compelling solution for transparent displays^3^. Basic research employs simple transparent OLED (TrOLED) devices with transparent anode and cathode, emitting lights from both sides simultaneously, without thin film transistors (TFTs) and driving circuits^4^. However, in commercial TrOLED displays, opaque driving circuits restrict light emission to only one side. This occurs because an opaque pixel electrode, typically positioned on the driving circuit, emits light, while areas without a pixel electrode remain non-emissive, ensuring transparency. Consequently, commercially available transparent displays are designed to be viewable from one side^5^.

Transparent displays impose constraints on personal information security and user concentration. This makes TrOLEDs suitable primarily for public use or facilitating communication between individuals on opposite sides. However, conventional TrOLEDs displaying images on only one side have clear limitations for the purposes. Therefore, bidirectional transparent displays, capable of providing different information on each side and selectively turning on or off, are highly sought after to broaden their applicability in various circumstances. Despite their potential, achieving bidirectional transparent displays with dual-viewing capabilities remains a significant challenge and has yet to be demonstrated.

Top-emission structures are typically employed in unidirectional TrOLEDs, owing to the opaque driving circuits on the backplane substrate^6^. A transparent cathode material is essential for both bidirectional and top-emission unidirectional TrOLEDs. Indium tin oxide (ITO), a well-known transparent electrode material, is typically used as a pixel anode because of its large work function (4.7 eV) and requirement for sputtering processes; however, it is not suitable for cathode electrodes^7,8^.

Ultrathin metal films are promising candidates for transparent cathode materials, in which a thin metal layer, such as Ag, is deposited at a thickness typically below 20 nm to ensure transparency^9,10^. However, Ag tends to form islands during thin-film deposition due to its surface kinetic instability, even at room temperature, making the fabrication of a thin continuous Ag film challenging^11^. The formation of island structures leads to high sheet resistance and optical loss, complicating the achievement of both high transmittance and low sheet resistance with thin Ag films.

Various methods have been employed to address this issue. A seed layer with high surface energy can mitigate the surface diffusion of Ag, facilitating the formation of a continuous film^12,13^. Another approach involves doping another metal into the Ag film to reduce the diffusion rate of Ag adatoms, leading to higher nucleation densities and smaller particle sizes, thus producing a thin and continuous Ag alloy film. However, choosing an appropriate dopant metal for a transparent electrode is not simple. Aluminum (Al)^14^, nickel (Ni)^15^, and copper (Cu)^15^ have high work functions, making their alloys with Ag unsuitable for cathodes; for instance, Ag:Ni and Ag:Cu alloys are typically used as anodes rather than cathodes. Magnesium (Mg)^16^ and calcium (Ca)^17^ can reduce optical transmittance owing to their high absorption coefficients.

In this study, we combined the seed layer concept and the metal doping mechanism to obtain a highly uniform Ag alloy layer. We selected zinc oxide (ZnO) as the seed layer to mitigate the surface diffusion of Ag. In addition, ZnO also improved charge injection ability when in contact with electrodes with small work functions by creating degenerated regions^18,19^. Ytterbium (Yb) was co-deposited with Ag as a doping material. The Yb:Ag film on ZnO exhibited an ultrasmooth surface morphology. Additionally, thin and uniform Yb:Ag films exhibit high transmittance in the visible light range and have low and tunable work functions^20–22^. Consequently, the performance of the TrOLED device with the ZnO/Yb:Ag cathode was excellent. Furthermore, we explored the potential of TrOLEDs for bidirectional viewing, with different information displayed on each side and the capability to selectively turn the rear-side viewing on or off.

## Results

Transparent and thin metal cathodes typically incorporate a capping layer (CPL) to enhance their optical transmittance and protect the underneath layers^23^. In our study, *N*,*N*′-Di(1-naphthyl)-*N*,*N*′-diphenyl-(1,1′-biphenyl)-4,4′-diamine (NPB) was used as the CPL, and a ZnO layer was used as the seed layer. The entire cathode unit including a CPL and electron injection layer and electron transport layer consisted of bathophenanthroline (Bphen)/ Bphen:Liq/ ZnO/ Yb:Ag/ NPB from the bottom, as described in Fig. S1a.

**Fig. 1:**
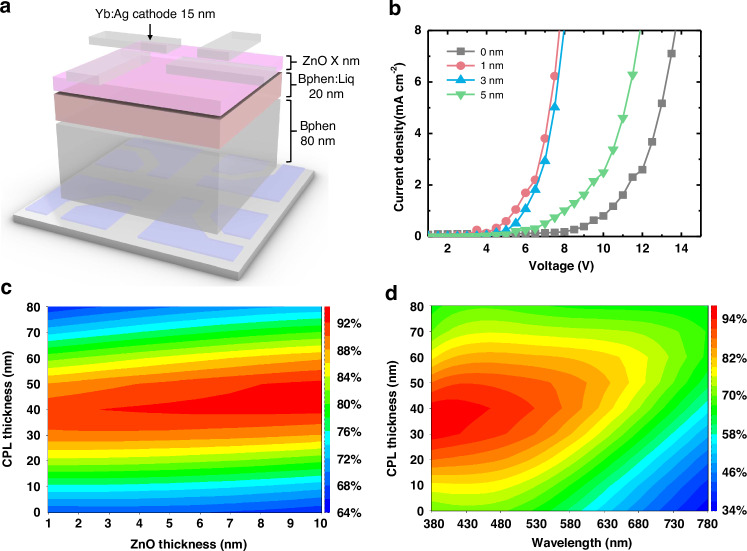
Optimization of the ZnO and capping layer (CPL) thicknesses in the cathode unit. **a** An electron only device (EOD) including a cathode unit, electron injection layer (Bphen:Liq) and electron transport layer (Bphen), but without a CPL. **b** Current density of the EODs with varying ZnO thicknesses. **c** Simulation results for the optical transmittance of a cathode unit with varying ZnO and CPL thicknesses at a wavelength of 510 nm. Color represents the optical transmittance. **d** Optical simulation of the entire cathode unit with CPL thickness in the entire visible wavelength region at a fixed ZnO thickness of 3 nm

The thickness of the ZnO layer significantly affected the charge injection capability of the cathode unit. To optimize the ZnO layer thickness, an electron-only device (EOD) with varying ZnO thicknesses (0 nm, 1 nm, 3 nm, and 5 nm), in which the CPL was excluded (Fig. 1a), was fabricated, and their current densities were measured. As shown in Fig. 1b, the current density was the highest when the ZnO thickness was between 1 nm and 3 nm. However, when the ZnO thickness increased to 5 nm, the current density decreased sharply. When the ZnO layer is sufficiently thin (1–3 nm), the interface between ZnO and the organic layer is included in a strongly degenerate region induced by the metal electrode, and as a result, the electrode unit has a high carrier density.^24,25^ On the other hand, when the ZnO layer is thicker (>5 nm), the interface between the ZnO and the organic layer is in a weakly degenerate region, in which the carrier density is relatively low. Therefore, when the metal layer and the interface between the ZnO and organic layers are close, that is, when the ZnO thickness is between 1 nm and 3 nm, the electrical properties of the cathode unit increase dramatically (see Fig. S1 for the details of the degenerate region.)

Simulations indicated that the optical transmittance was strongly influenced by the CPL thickness but also showed a weak dependence on the ZnO thickness (Fig. 1c). At a fixed CPL thickness of 40 nm, the optical transmittance gradually increased with the increasing ZnO thickness. This occurs because the ZnO layer, with its high refractive index, can function as an antireflection coating on the Yb:Ag metal layer, reducing reflection and enhancing transmittance. By balancing the electrical properties (Fig. 1b) with the optical transmittance (Fig. 1c), the optimal ZnO thickness was determined to be 3 nm.

The optimal CPL thickness was determined based on the optical transmittance at 510 nm and across the entire visible wavelength range, as well (Figs. 1d and S2). In our device, 510 nm was selected as the characteristic wavelength because the emission layer (EML) exhibited the peak wavelength at 510 nm, as shown in Fig. S3. As depicted in Figs. 1b, c and S2b, the highest average optical transmittance in the visible spectrum, as well as at 510 nm, was achieved with CPL thicknesses between 30 nm and 50 nm. Therefore, the thickness of the CPL was set to 40 nm.

**Fig. 2:**
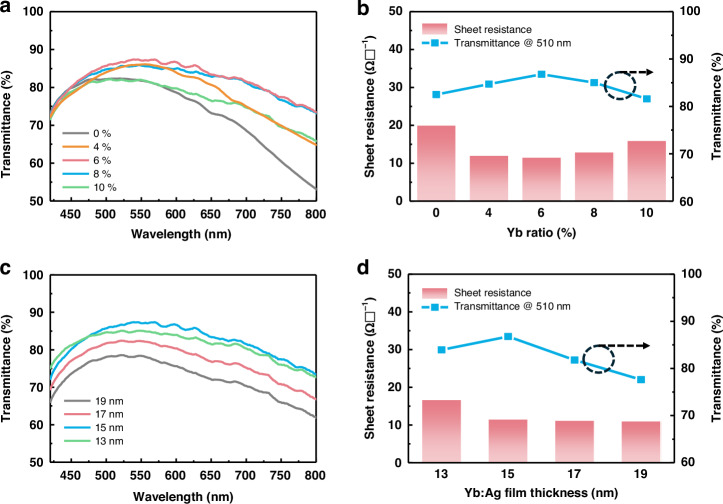
Optimization of the optical and electrical properties of Yb:Ag films. **a** Optical transmittance of 15-nm-thick Yb:Ag films with varying Yb doping ratios. **b** Sheet resistance and optical transmission at 510 nm of the 15-nm-thick Yb:Ag films as a function of the Yb doping ratio. **c** Optical transmittance of 6% Yb:Ag films with varying thickness. **d** Sheet resistance and optical transmission at 510 nm of 6% Yb:Ag films with varying thickness

Subsequently, the optical transmittance and sheet resistance were measured for various doping ratios and thicknesses of the Yb-doped Ag layers. As shown in Fig. 2a, a 15-nm pure Ag film deposited without Yb dopant exhibited an average transmittance of only 58%, with a sharp decrease in transmittance occurring particularly at long wavelengths. The Yb-doped Ag films significantly improved the transmittance at longer wavelengths. In particular, when the Yb doping ratio was 6–8%, the optical transmittance at 510 nm exceeded 85%, and that at longer wavelengths increased more than 25%. The optical loss is known to be related to the imaginary part of optical permittivity (ε”)^26^. When the Yb doping ratio is lower than the optimal range of 6–8%, the free electrons are largely scattered due to the rough surface morphology, causing a large ε” and optical loss. As the Yb doping ratio increases to 6–8%, the surface becomes even, and ε” and optical loss is reduced. When the Yb concentration exceeds the optimal range, the doped Yb atoms introduce additional electron scattering, causing extra loss for free electrons and optical loss. In addition, the sheet resistance of the Yb:Ag film was also minimized at a 6% Yb doping ratio, which is 41% smaller than that of a pure Ag film (Fig. 2b).

The figures of merit (FOMs) of the electrodes were derived by calculating the rate of transmittance at 510 nm with respect to the sheet resistance^27^. The FOM was calculated following Haack’s method, where it is defined as *T*^10^/*R*ϒ [Ω^−1^], with *T* representing the optical transmittance and *R*ϒ the sheet resistance. The Yb:Ag film with a 6% doping ratio exhibited the highest FOM, as shown in Table 1. Thus, the Yb doping ratio was set to 6% in the remaining experiments. Then, the optical and electrical performances of the Yb:Ag film were examined by varying the film thickness at a fixed Yb-doping ratio of 6%, and the results are shown in Fig. 2c, d. As the thickness decreased from 19 nm to 15 nm, the transmittance gradually increased, whereas the sheet resistance remained relatively unchanged. However, when the Yb:Ag film thickness decreased further to 13 nm, the optical transmittance decreased and the sheet resistance abruptly increased. As the film became thinner, the optical loss caused by light scattering increased owing to increased surface roughness. This can lead to a decrease in light transmission^28,29^. Thus, the optimal FOM was observed at a 6% Yb doping ratio and 15 nm thickness, which were selected in our study for the fabrication of OLED devices (Table 2). To verify the optical Yb doping ratio at different Yb:Ag alloy film thickness, we carried out the same analyses for a 13-nm thick Yb:Ag alloy film, which exhibited the same optimal Yb doping ratio of 6%, as shown in Fig. S4 and Table S1.

**Table 1:** Figure of merit (FOM) of Yb:Ag electrodes with varying Yb ratio at 15 nm thickness (*T*_vis_ and *T*_510nm_ represent the average optical transmittance in the entire visible spectrum and the transmittance at 510 nm, respectively.)

Yb Doping concentration	*T*_vis_ [%]	*T*_510nm_ [%]	Sheet resistance [Ω ϒ^−1^]	FOM [×10^–3^ Ω^−1^]
10%	67.33	81.59	16	8.17
8%	73.31	85.02	12.96	15.23
6%	72.95	86.78	11.61	20.86
4%	65.57	84.70	12.11	15.69
0%	58.59	82.5	20.07	7.28

**Table 2:** FOM of Yb:Ag electrodes with varying thickness at a 6% Yb ratio

Thickness	*T*_vis_ [%]	*T*_510nm_ [%]	Sheet resistance [Ω ϒ^−1^]	FOM [×10^–3^ Ω^−1^]
13 nm	72.73	83.95	16.77	10.36
15 nm	72.95	86.78	11.61	20.86
17 nm	67.38	81.79	11.24	11.91
19 nm	62.31	77.61	11.08	7.16

The optical and electrical properties are closely related to the surface morphology, which is influenced by the surface energy of films. When a metal layer with a surface free energy of *Ym* is deposited on a seed layer with a surface free energy of *Ys*, the metal film can be formed as a continuous and uniform layer if the following surface condition is satisfied^30^:

1 $${\Upsilon }m+{\Upsilon }i \,<\, {\Upsilon }s$$

where, *Υi* represents the interfacial free energy between the metal and substrate.

**Fig. 3:**
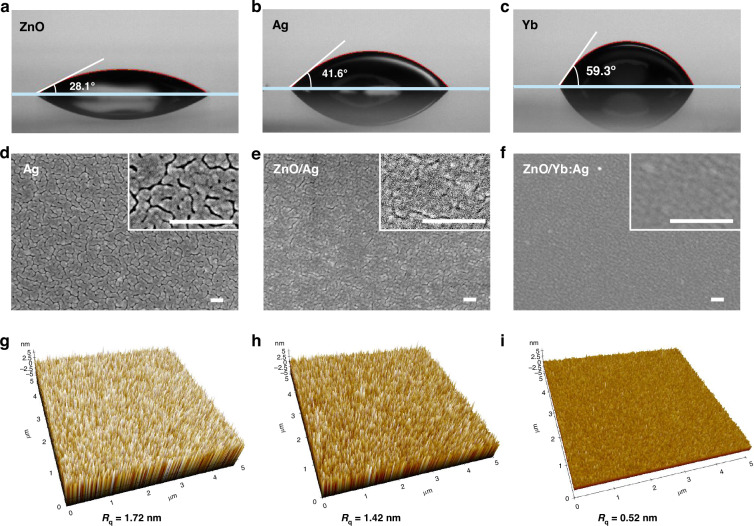
Surface energy and morphology of ZnO, Ag, ZnO/Ag, and ZnO/Yb:Ag films. **a**–**c** Contact angles of a water droplet on ZnO, Ag, and Yb films, respectively. The contact angle is inversely proportional to the surface energy. **d**–**f** Scanning electron microscopic (SEM) images of 15-nm-thick Ag (**a**), ZnO/Ag (**b**), and ZnO/Yb doped Ag films (**c**). All scale bars, 100 nm. **g**–**i** Atomic force microscopic (AFM) images and surface roughness of 15-nm-thick Ag (**d**), ZnO/Ag (**e**), and ZnO/Yb doped Ag films (**f**)

The surface contact angles of the seed layer and thin metal film were analyzed, as shown in Fig. 3a–c. When a water droplet was dropped onto the ZnO film, the contact angle was small (28.1°), which was calculated to have a surface energy of 64.47 mJ m^−2^ using the Good and Girifalco model^31^. The electron injection material, Bphen, is known to have a relatively high contact angle (about 65°) and low surface energy^32^ Therefore, the ZnO seed layer can effectively increase the surface energy underneath the cathode. The water contact angles for the Ag and Yb layers were 41.6° and 59.3°, respectively, indicating that the Ag and Yb layers have relatively lower surface energies than ZnO. The surface energies of Ag and Yb were calculated to be 55.60 mJ m^−^^2^ and 41.53 mJ m^−^^2^, respectively. A material with a high surface energy is suitable for a seed layer because it tends to enhance the uniformity of a deposited material with lower surface energy, according to Eq. (1). Therefore, a ZnO seed layer with high surface energy is suitable for the deposition of Ag and Yb layers. However, Ag with higher surface energy may have a higher surface diffusion rate than Yb, causing partial aggregation of Ag on ZnO when very thin. On the other hand, co-deposited Yb atoms with lower surface energy have a lower surface diffusion rate and even can impede the surface diffusion of Ag atoms, enhancing the film uniformity.

To verify the effect of Yb atoms on Ag alloy film formation, the surface morphologies of three types of electrodes, Ag, ZnO/Ag, and ZnO/Yb:Ag films on Bphen:Liq layer, were examined. Figure 3d, i shows the scanning electron microscopic (SEM) and atomic force microscopic (AFM) images of the three surfaces. When pure Ag was deposited on a substrate with a 20-nm Bphen:Liq 20-nm layer on a silicon wafer, the grain boundaries were clearly discernible in the SEM image (Fig. 3d). The aggregation of Ag adatoms arises from the strong surface diffusion of Ag on Bphen:Liq, which can be explained by the Volmer-Weber growth model^33,34^. When the thickness of the Ag film was less than 20 nm, it failed to form a continuous thin film; instead, island-type Ag aggregation was observed, as shown in the SEM image in Fig. 3d. The Ag layer deposited on ZnO (Fig. 3e) exhibited slightly diminished grains because of the ZnO seed layer; however, the grains were still clearly discernible. The surface energy of the ZnO layer (*Ys* in Eq. (1)) was not sufficiently high to completely suppress the surface diffusion of Ag, resulting in a partially aggregated Ag film. In contrast, a 15-nm-thick Yb:Ag film on ZnO shows a uniform surface without clear grains at the same magnification (Fig. 3f). Interestingly, this confirms that the Yb atoms (at only 6%) in the ZnO/Yb:Ag film can effectively suppress the surface diffusion of the Ag atoms that comprise 94% of the Yb:Ag film.

The same results were observed in the AFM analysis. The surface roughness (Rq) of pure Ag on Bphen:Liq was 1.72 nm (Fig. 3g), indicating an irregular surface morphology caused by the strong surface diffusion and aggregation of Ag adatoms. The pure Ag layer deposited on ZnO exhibited a slight reduction in surface roughness (Rq = 1.42 nm, Fig. 3h). The surface roughness of a Yb:Ag film on ZnO was surprisingly low of Rq = 0.52 nm, as shown in Fig. 3i. Thus, a small portion of Yg atoms dramatically suppressed Ag surface diffusion and improved the surface morphology.

**Fig. 4:**
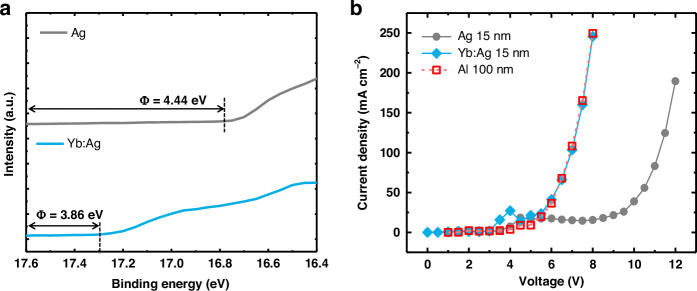
Comparison of electrochemical properties of pure Ag and Yb:Ag films. **a** Work function of Ag and Yb:Ag electrodes. **b** Current vs voltage (*I*–*V*) characteristics of cathode units with various metal materials

The work functions of the metal films were analyzed using ultraviolet photoelectron spectroscopy (UPS), as shown in Fig. 4a. According to the UPS analysis, the Yb-doped Ag film had a lower work function than pure Ag; the work function of Ag was approximately 4.44 eV, and that of the Yb:Ag film was approximately 3.86 eV, exhibiting a decrease of approximately 0.6 eV. This reduction is attributed to the low work function of pure Yb (2.6–2.8 eV). A reduction in the work function can enhance the electron injection in OLEDs, which is highly desirable but challenging.

The electron injection ability of each cathode was examined by measuring the electrical characteristics of the EOD, whose structure is shown in Fig. S5. As shown in Fig. 4b, the current density is significantly enhanced by doping the Ag electrode with 6% Yb. The inferior electron injection of the pure Ag electrode is caused by the high sheet resistance and relatively large work function of the Ag layer. In contrast, a 15-nm-thick Yb:Ag electrode exhibited a significantly enhanced current density and electron injection ability comparable to that of a 100-nm-thick Al layer, as depicted in Fig. 4b. Thus, the improved surface morphology and surface electron injection ability of the Yb:Ag film significantly enhance the electrical performance of the cathode. As a result, a thin 15-nm Yb:Ag film may replace a thick Al cathode usually used in conventional OLEDs. When comparing the proposed Yb:Ag cathode with other metal-based transparent cathodes reported to date, it may not be the best in every individual property but demonstrates outstanding overall performance without any significant weakness, as shown in Table S2. In particular, it ranks among the lowest in surface roughness and features a tunable work function within a range suitable for cathode electrodes.

**Fig. 5:**
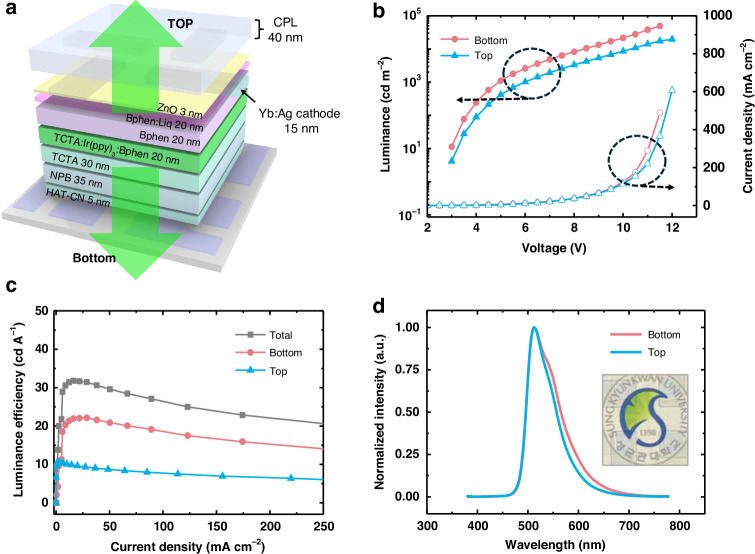
Electro-optical performance of TrOLEDs with a Yb:Ag cathode. **a** Layer structure of TrOLEDs with Yb:Ag cathode electrode, and **b** J-V-L characteristics. **c** Luminous efficiency vs. current density, and **d** electroluminescence (EL) spectra of the TrOLED device

Simple TrOLEDs devices were fabricated using Yb-doped Ag electrodes. Figure 5a shows the layered structure of the fabricated device. The bottom anode was an ITO electrode, and the top cathode was a 15-nm, 6% Yb-doped Ag electrode. As shown in Fig. 5b, c, the fabricated TrOLED exhibited excellent luminance, exceeding 10,000 cd m^−2^ on both sides. However, there was a significant imbalance in the luminance and device efficiency on the top and bottom sides. The luminance at the bottom was approximately twice that at the top. This asymmetric luminance is caused by the different optical transmittance and reflectance of the ITO anode and Yb:Ag cathode (see Fig. S6). However, the luminance difference was much greater than the variation in light transmittance between the two electrodes. The enhanced luminance difference results from multi-reflection and interference effects within the optical cavity containing heterogeneous mirrors, as confirmed through optical simulation (Figs. S7 and S8)^35–37^. The external quantum efficiency (EQE) is presented in Fig. S9. The efficiency is slightly lower than the bottom emission device reported in our previous study^21^. This may be due to the suboptimal cavity design in this device. Optimizing the cavity for bidirectional viewing devices remains a task for future work.

Figure 5d shows the emission spectra of the top and bottom layers. While the main emission peak was the same for both sides at 510 nm, a slight difference was observed in the shoulder peak owing to the cavity effect on the top side. Thus, the asymmetric cavity effect is related to the significantly different luminance on both sides^38–41^. The inset in Fig. 5d shows the transparency of the cathode unit; the text and images behind the OLED device are clearly readable. Therefore, the Yb:Ag electrodes can be used as cathode materials in TrOLEDs.

**Fig. 6:**
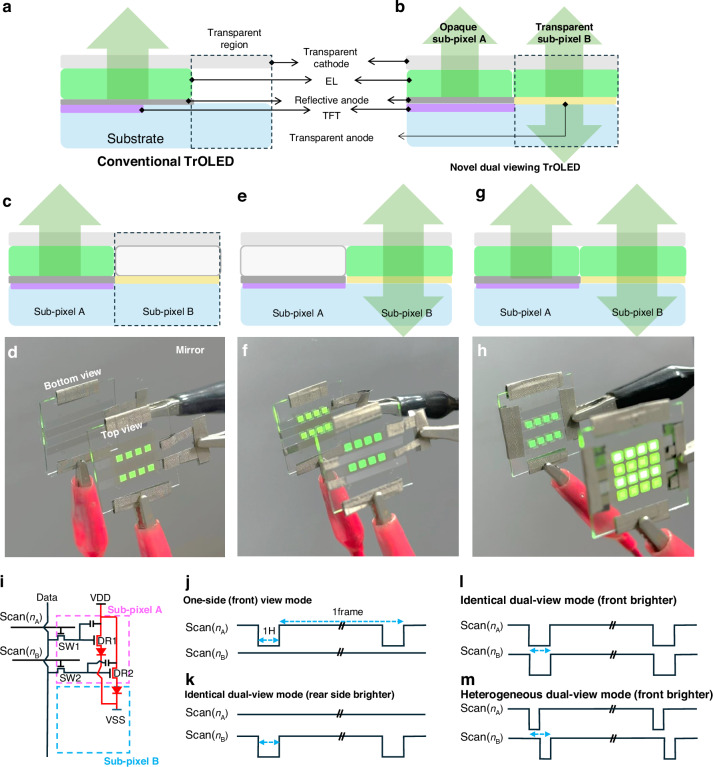
Driving schemes of the proposed bidirectional viewing TrOLED. **a**, **b** Comparison of conventional TrOLED display with one-side viewing and novel TrOLED display with bidirectional viewing. **c**, **d** Pixel structure and photographs in the one-side-view mode, **e**, **f** identical dual-view mode, and **g**, **h** identical or augmented dual-view mode, respectively. A mirror was placed behind the device to observe the rearview. **i** Equivalent circuit for a pixel of novel TrOLED. **j**–**m** Scan signals to correspond to four types of display modes. Here, k and l are identical dual view modes but have different brighter side

In real applications, TrOLED devices usually adopt a top-emission structure with a non-emissive transparent area, as shown in Fig. 6a, where a reflection layer is located on top of the driving circuit on the bottom substrate and the transparent region does not have an EML layer. Therefore, the emission areas on the reflection layer and transparent areas are contradictory in the conventional TrOLEDs. In addition, conventional TrOLEDs display information on only one side.

Herein, we propose a TrOLED display with bidirectional viewing (Fig. 6b), in which a pixel is composed of two sub-pixels: sub-pixel A with a reflector on the bottom driving circuit area and sub-pixel B in the transparent region. Sub-pixels A and B have separate pixel anodes and driving circuits and are independently controllable. Subpixel A emits light toward the top side only, whereas subpixel B emits light simultaneously toward both sides. To emulate the proposed novel TrOLEDs, we fabricated a simple TrOLED device with 4 × 4 pixels, in which the pixels in the even (second and fourth) rows were sub-pixels A with a reflective anode, and those in the odd (first and third) rows were sub-pixels B with a transparent anode.

The proposed TrOLEDs can be operated in three distinct modes: one-side view, identical dual view, and augmented dual view. In the one-side-view mode (Fig. 6c), only sub-pixels A are activated, resulting in an image visible only on the front side. Figure 6d shows a photograph of the 4 × 4 TrOLED device in the one-side-view mode, where only the even rows are turned on. A mirror placed behind the device confirms that the image is not visible from the rear side, as evidenced by Fig. 6d. Figure 6e, f demonstrates the identical dual-view mode, where only sub-pixels B are turned on. As depicted in Fig. 6f, the displayed images are clearly visible on both sides and are identical, apart from the mirror inversion. When both sub-pixels A and B are activated as shown in Fig. 6g, h, the displayed image can be either in identical or augmented dual-view mode. As shown in Fig. 6h, the displayed image is visible on both sides, but the front-side image has more pixels visible. If the data on sub-pixel As are equal to that on sub-pixel Bs, both front and rear views are identical. However, if the data on sub-pixel As are different from those on sub-pixel Bs, the front-view contains more information than the rear-side image. The luminance levels in the front and rear sides are different even in the identical dual-view mode. In the identical dual-view mode using sub-pixel Bs (Fig. 6e, f), the real-view is brighter due to the different optical properties of the anode and cathode electrodes. However, when the sub-pixel As is turned on, as shown in Fig. 6g, h, the front view is much brighter. Additional operational examples are provided in Movie S1.

Although the dual-view TrOLED modes shown in Fig. 6c–h are emulated using a simple device without a TFT circuit, the corresponding pixel structure in real display panels can be easily developed. An example of pixel driving circuits for the dual-view TrOLED is illustrated in Fig. 6i. Sub-pixels A and B are connected to driving TFT 1 (DR1) and 2 (DR2), respectively. Sub-pixel A has a reflective anode such as ITO/Ag/ITO, and sub-pixel B has a transparent anode. The data signals of these sub-pixels are provided through switching TFT 1 (SW1) and 2 (SW2) by controlling the scan signals Scan(n_A_) and Scan(n_B_), respectively. Thus, sub-pixels A and B can be selectively turned on or off by applying the corresponding signal through the Scan(n_A_) or Scan(n_B_) electrode. Figures 6j–m depict the scan signals required to activate three different modes. For the one-side view mode, only Scan(n_A_) signals are activated while the Scan(n_B_) signals remain off (Fig. 6j). Both of Fig. 6k, l represent identical dual-view modes, but with different brightness levels. When only sub-pixel B is turned on, the rear side displays a brighter image (Fig. 6k). However, when both sub-pixels A and B are simultaneously activated, the same images appear on both sides, with the front side being much brighter. To enable the heterogeneous dual-view mode, different image data must be written for sub-pixels A and B. This is achieved by dividing a single scan on time (1H) into two halves, with different data signals written on each sub-pixel, as illustrated in Fig. 6m.

## Discussion

We developed a highly transparent Ag-based cathode with excellent surface properties using a ZnO seed layer and Yb doping to fabricate transparent OLEDs. The high-surface-energy ZnO seed layer and the low-surface-energy Yb doping resulted in the ideal growth of the Yb:Ag thin film, achieving a very smooth surface with an Rq of 0.52 nm at a thin 15 nm film. The electrode exhibited a low sheet resistance of 11.6 Ω ϒ^−1^ and an excellent optical transmittance of 86.7% at 510 nm. It also reduced the energy barrier for electrons to be injected into the organic layers by adjusting the work function to 3.86 eV. The work function can be adjusted by modifying the doping ratio of Ag with a high work function and Yb with a low work function. The proposed Yb-doped Ag films can effectively replace reflective electrodes in conventional flat-panel displays because of their low surface roughness, high transmittance, and low injection barriers.

In addition, we proposed a TrOLED with selective bidirectional viewing that can operate in three different modes. The three viewing modes of the TrOLED device with a novel pixel structure can be used under various user scenarios by selectively displaying images on subpixels A and B. For example, this type of display can be used for counseling or teaching purposes; the front-side user can share a part of the information with the rear-side user while keeping some information hidden. As another example, the front-side image could be displayed brighter than the rear-side image; this type of display is commonly required when the display is placed in the window of a building or automobile, where the inside is darker than the outside (or vice versa).

However, bidirectional viewing TrOLED technology is still in its early stage and requires significant effort to be applied to real products. One challenge is achieving simultaneous white balance adjustment on both sides. In conventional one-sided OLEDs, white balance is managed by controlling the relative luminance of the three primary colors. However, this is more difficult with bidirectional OLEDs due to differences in spectral efficiency on each side. Despite this challenge, the novel TrOLEDs have the potential to greatly expand the applicability of transparent displays across various user scenarios.

## Materials and methods

### Materials

The 1,4,5,8,9,11-hexaazatriphenylene hexacarbonitrile (HAT-CN) and NPB were procured from Ossila, UK. 4’,4”-tri(*N*-carbazolyl)triphenylamine (TCTA), Bphen, tris(2-phenylpyridine)iridium(III) (Ir(ppy)3), and 8-quinolinolato lithium (Liq) were obtained from Javachem, South Korea. Zinc oxide (1–4mmpcs, 4N5), ytterbium (1–4 mm pcs, 4 N), and silver (3–5 mm granules, 4 N) were sourced from Tasco, South Korea. These materials were deposited on the substrates without further purification by thermal sublimation in a vacuum chamber.

### Device fabrication characterization

All devices were fabricated on a glass with patterned indium tin oxide (ITO) serving as the anode, bare glass, or silicon substrate. The substrates were cleaned with ethanol and deionized water using sonication for 10 min each, followed by the removal of residual solvents on a hotplate at 150 °C. Oxygen (O_2_) plasma treatment was conducted under high vacuum conditions of 5.0 × 10^–6^ Torr or less. Organic and metal materials for the device fabrication were deposited using the evaporation equipment in separate organic and metal chambers under identical high-vacuum conditions. ZnO was also deposited in the metal chamber of the evaporation equipment, and its evaporation conditions include the *Z*-factor of 1.080 and tool factor of 33.5%. The film characteristics of Bphen:Liq and ZnO are shown in Fig. S10. The OLED device consisted of ITO(150 nm)/HAT-CN(5 nm)/NPB(35 nm)/TCTA(30 nm)/TCTA:Bphen:Ir(ppy)3(20 nm)/Bphen(20 nm)/Bphen:Liq(20 nm)/ZnO(3 nm)/Yb:Ag(6%, 15 nm)/NPB(40 nm). The Yb-doped Ag films were co-evaporated on the substrates or OLED devices. The Yb:Ag alloy ratio was controlled by changing the deposition rate. For 10% doping, the Yb:Ag deposition rate is 0.2:2 Å s^−1^; for 8%, it is 0.16:2 Å s^−^^1^; and for 6%, it is 0.14:2 Å s^−^^1^. The organic and metal layers were deposited using a thermal evaporator (SUNICEL Plus 200, Sunic System, Korea).

### Device characterization

Optical transmittance simulations were conducted using TechWiz OLED (Sanayi Systems, Korea). To measure the optical transmittance and sheet resistance of the cathode unit, Bphen (20 nm)/Bphen:Liq (20 nm)/ZnO (3 nm)/Yb:Ag (x%, varying thickness)/NPB (40 nm) were sequentially deposited on a glass substrate. The optical transmittance spectra of the cathode units were measured using a UV-vis spectrophotometer (AvaSpec-2048L, Avantes, Netherlands). The sheet resistance of the cathode was measured using a 4-point probe station (CMT-100MP, Atlas Copco, Sweden). For the surface energy calculations, water was dropped onto ZnO (50 nm), Ag (50 nm), and Yb (50 nm) samples deposited on glass substrates, and the contact angle was measured using a contact angle meter (Smart Drop Plus, Femtobiomed, Korea). For the surface morphology analysis, three types of samples were prepared: Bphen:Liq (20 nm)/Ag (15 nm), Bphen:Liq (20 nm)/ZnO (3 nm)/Ag (15 nm), and Bphen:Liq (20 nm)/ZnO (3 nm)/Yb:Ag (6%, 15 nm) were deposited on silicon substrates. The surface morphology of the cathode was analyzed using scanning electron microscopy (SEM; JSM-7600F, JEOL, Japan) and atomic force microscopy (AFM; *n* × 10, Park Systems, Korea). For surface potential energy analysis, 150 nm of ITO was deposited on a silicon substrate, followed by Ag (15 nm) and Yb:Ag (6%, 15 nm). The surface potential energy was measured using ultraviolet photoelectron spectroscopy (UPS; NEXSA, Thermo Fisher Scientific, USA). The surface chemical composition and the chemical state were characterized using an X-ray photoelectron spectroscope (XPS; NEXSA, Thermo Fisher Scientific, USA). The current density–voltage–luminance (*J*-*V*-*L*) characteristics and electroluminescence spectra of the TrOLED devices were measured using a spectroradiometer (CS-2000, Konica Minolta, Japan) and an electrometer (Keithley 2400, Tektronix, USA).

## Supplementary information

Supplementary Information

Supplementary movie

## Data Availability

The data underlying the results presented in this paper are available from the corresponding author upon reasonable request.
