# Supplementary material for: Transparent OLED displays for selective bidirectional viewing using ZnO/Yb:Ag cathode with highly smooth and low-barrier surface
Source: Light Sci Appl. 2025 Jan 24;14:62. doi: 10.1038/s41377-024-01739-0 (PMC11759713; doi:10.1038/s41377-024-01739-0)
Supplement: Supplementary file 1 — Supplementary Information [file 41377_2024_1739_MOESM1_ESM.pdf]

Supplementary Information for

Transparent OLED displays for selective bidirectional viewing using ZnO/Yb:Ag cathode with highly smooth and low-barrier surface

Eun-young Choi[1], Sung-Cheon Kang[1], Kanghoon Kim[2], Su-Hyeon Lee[1], Jeong-Beom Kim[1] and Jang-Kun Song[1,2,*]

[1] *Department of Electrical and Computer Engineering, Sungkyunkwan University, Suwon-si, Gyeonggi-do 16419, Republic of Korea*

[2] *Department of Semiconductor and Display Engineering, Sungkyunkwan University, Suwon-si, Gyeonggi-do 16419, Republic of Korea.*

*Corresponding author: (Tel) 82-31-299-4599, (Email) jk.song@skku.edu

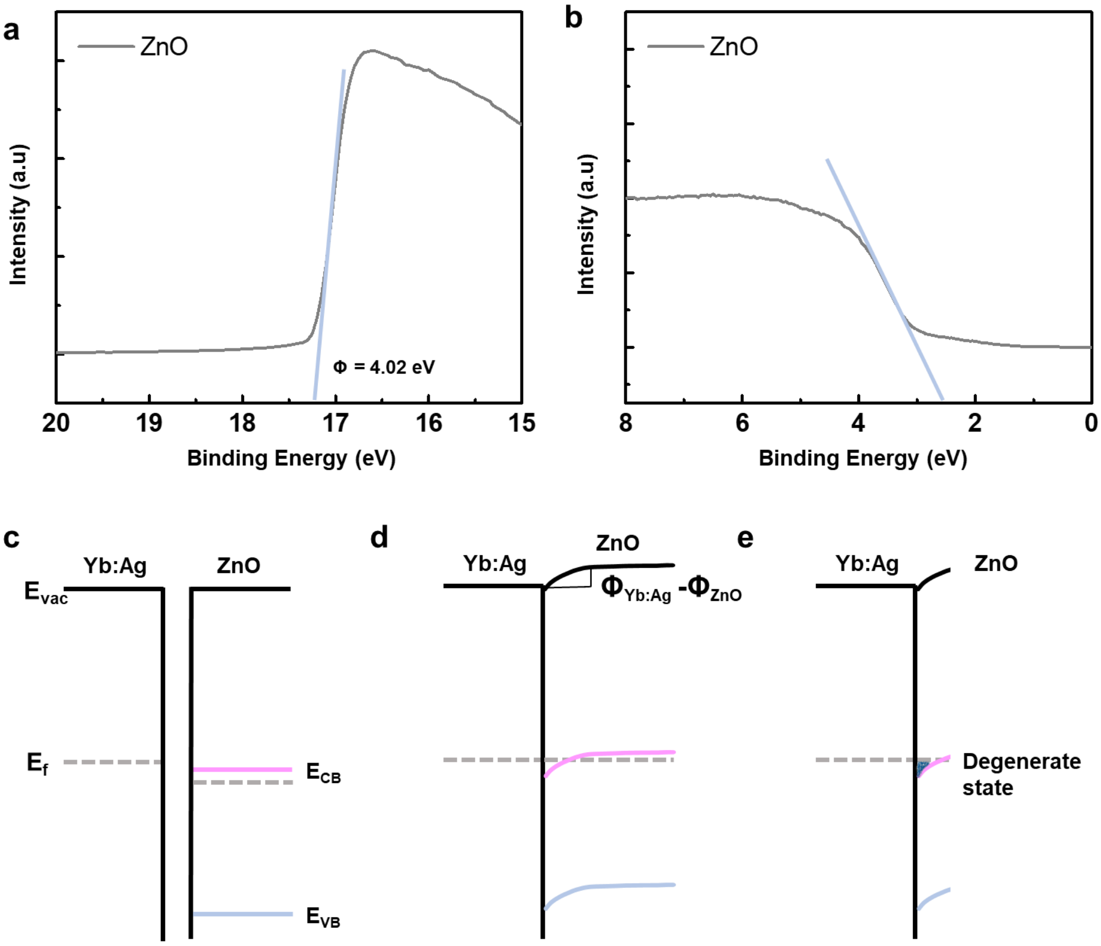

Supplementary Figure S1. a-b Secondary electron cut-off edges (a), and valence band edges (b) of a ZnO film fabricated by thermal evaporation. These were measured using ultraviolet photoelectron spectroscopy (UPS). **c-e** Energy level diagrams of Yb**:**Ag/ZnO before contact (c) and after contact (d) for thick ZnO case, and for ultra-thin ZnO layer (e), respectively.

The work functions of the Yb:Ag alloy film and thermally deposited ZnO film are 3.86 eV (Fig 4a) and 4.02 eV (Fig. S1a). When ZnO and the Yb:Ag alloy come into contact, the work function of the alloy is lower than that of ZnO. As a result, their Fermi levels align to the same energy level after reaching thermal equilibrium, resulting in band bending (Fig. S1d). By controlling the thickness of ZnO within the bending region, ZnO becomes degenerated (Fig. S1e). The region closer to the interface experiences a stronger degenerate state. As the distance from the interface increases, the material falls into a weaker degenerate region or exists outside the degenerate region.

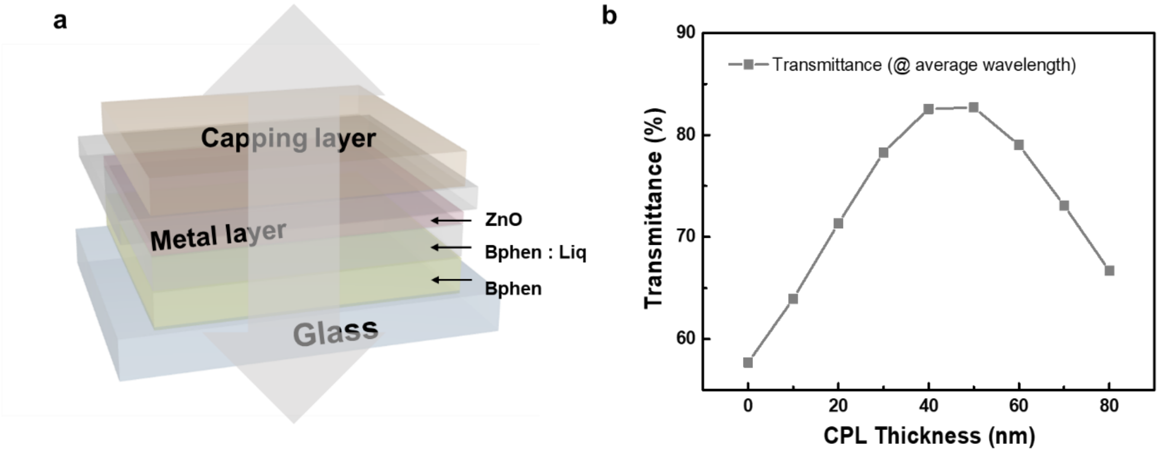

Supplementary Figure S2. a The layer structure of the entire cathode unit including a seed layer (ZnO) and a capping layer (NPB), which was fabricated for the optical measurement and simulation. The thicknesses of each layer were 20 nm for Bphen, 20 nm for Bphen:Liq, 3 nm for ZnO, and 15 nm for the Yb:Ag cathode. **b** The averaged optical transmittance in the visible wavelength region as a function of CPL thicknesses.

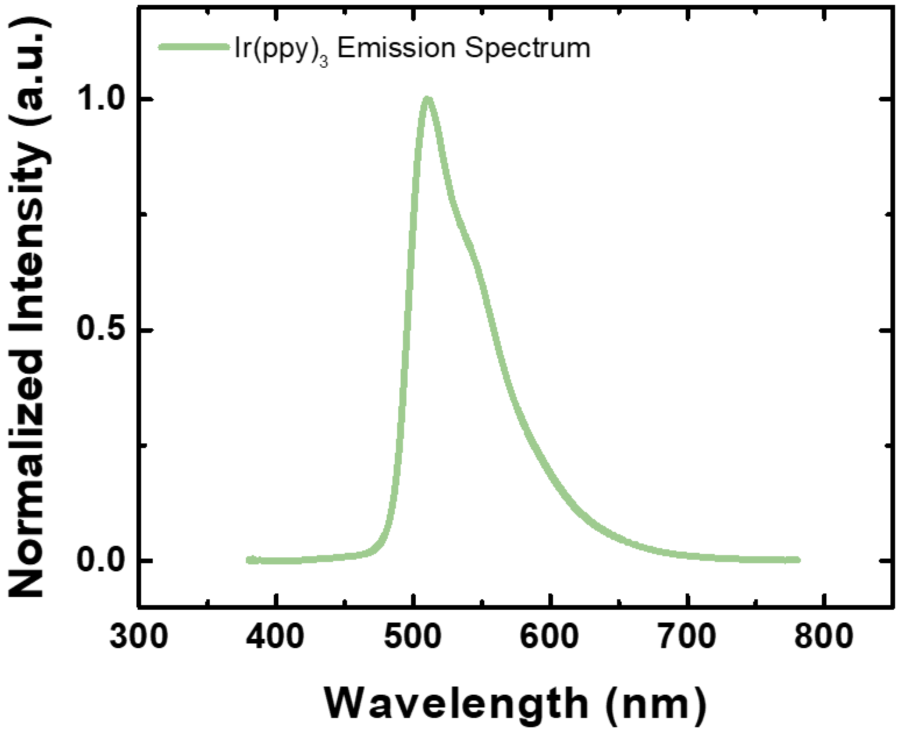

Supplementary Figure S3. The electroluminescence spectrum of the emission layer (TCTA:Bphen:$Ir(ppy)_3$) used in this study.

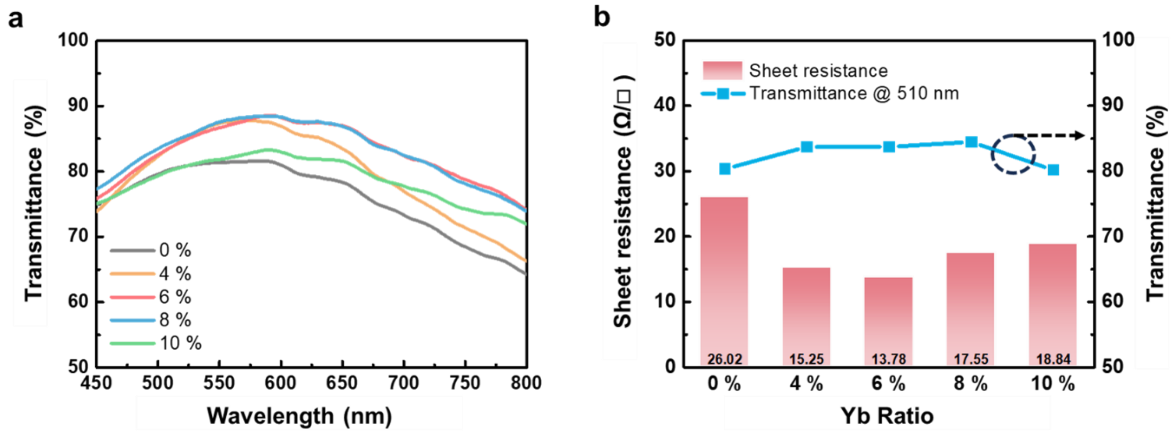

Supplementary Figure S4. a Optical transmittance of 13-nm-thick Yb:Ag films with varying Yb doping ratios. **b.** Sheet resistance and optical transmission at 510 nm of the 13-nm-thick Yb:Ag films as a function of the Yb doping ratio.

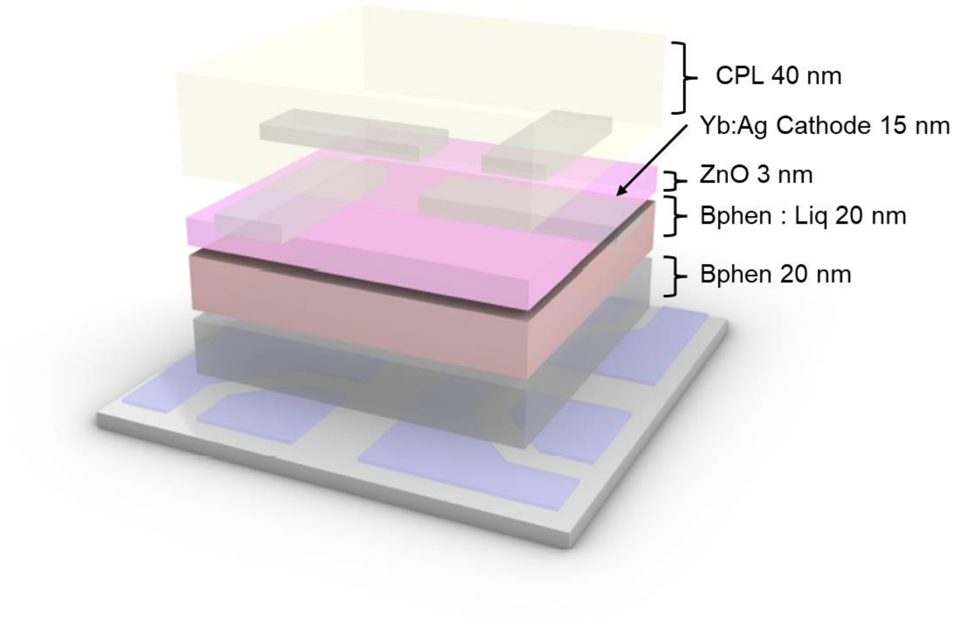

Supplementary Figure S5. Structure of an electronics-only device (EOD) for comparing current densities of different types of metal thin films in a cathode unit. The three EODs with different cathode materials were fabricated and evaluated to verify the electrical characteristics. The three metal cathode layers were Ag (15 nm), Yb:Ag (6%, 15 nm), and Al (100 nm), respectively.

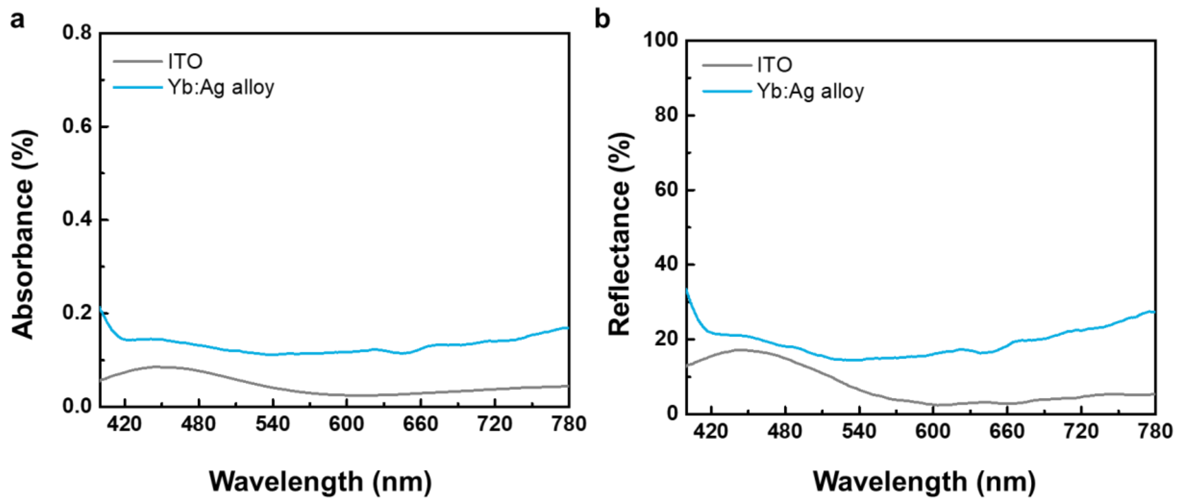

Supplementary Figure S6. a. Optical absorbance and **b.** optical reflectance of 15-nm, 6 % Yb:Ag cathode unit

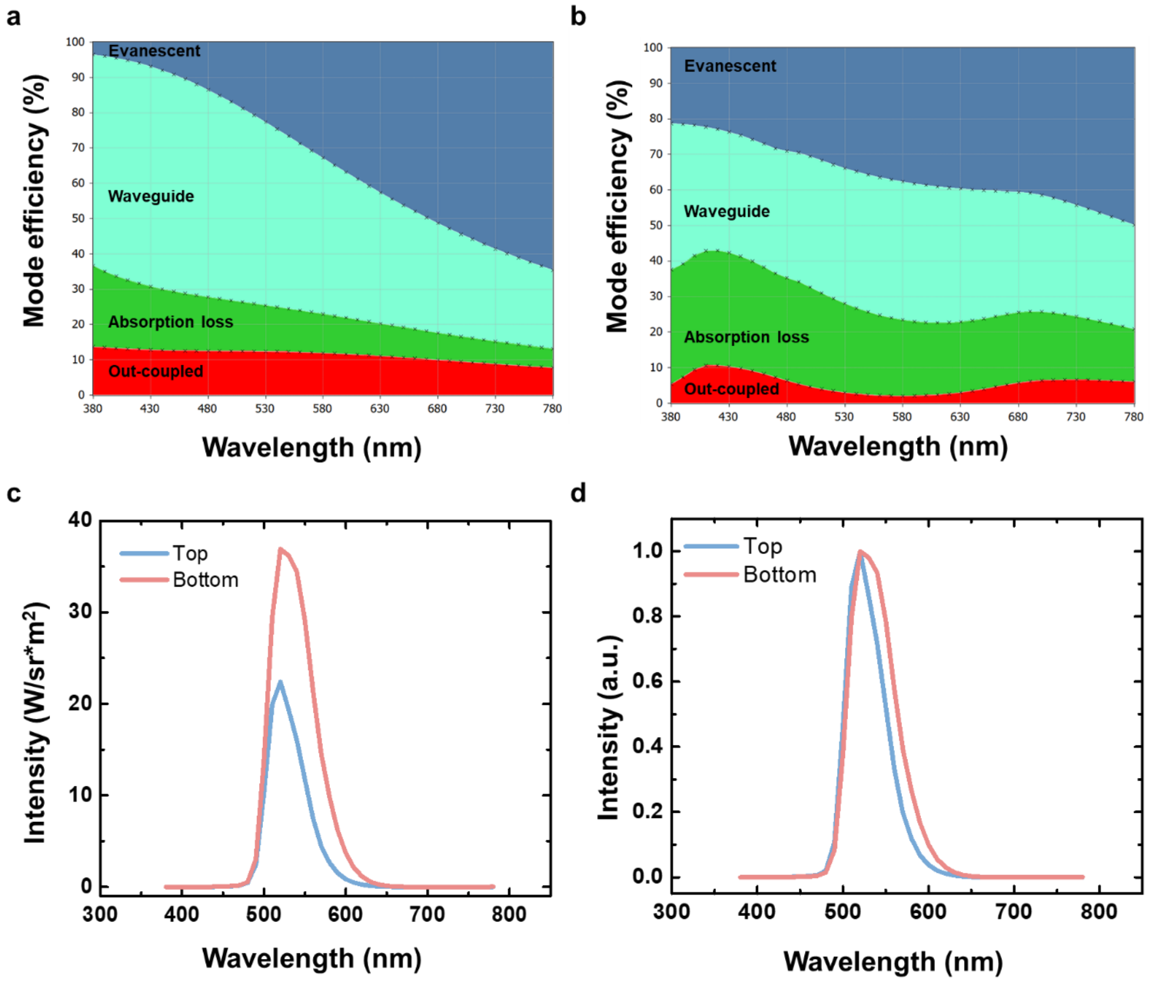

Supplementary Figure S7. a Optical simulation of mode efficiency for bottom emission, and **b** for top emission. **c** Optical simulation of spectral luminance profiles for bottom and top emissions. **d** Normalized luminance spectra and difference of spectral width for bottom and top emission.

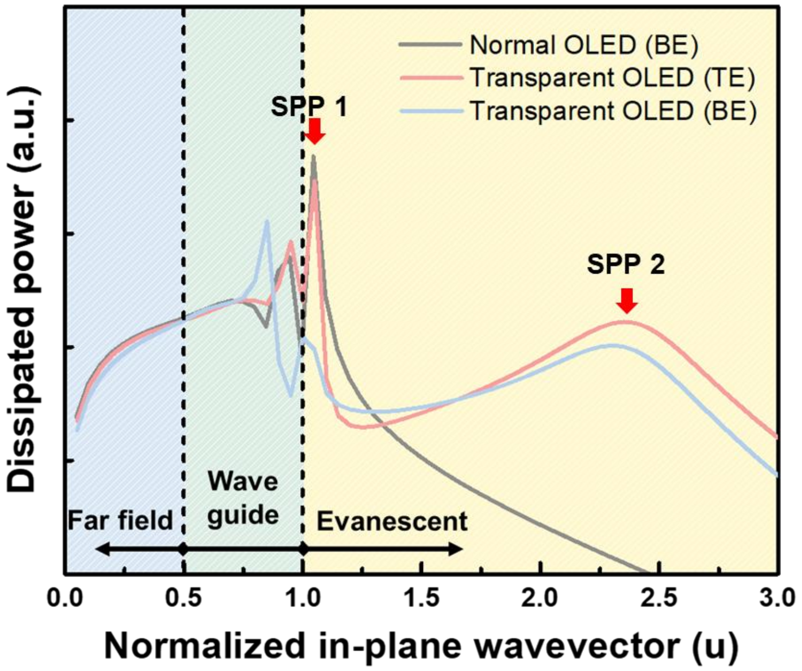

Supplementary Figure S8. Simulation for the comparison of dissipated power in a normal OLED with thick Al cathode and the transparent OLED used in this study. "BE" denotes bottom emission and "TE" denotes top emission.

Surface plasmon polaritons (SPPs) are electromagnetic wave that propagates along the interface between a metal and a dielectric or between a metal and air. They arise from the interaction between collective oscillations of free electrons in the metal and the electric field in the dielectric. In OLEDs, SPPs commonly form at the interfaces between metal electrodes and organic layers, where they interact with dipole radiation generated at these interfaces. In OLEDs, the dipole radiation can be coupled with SPPs at multi-layer interfaces such as metal/organic/air structures. However, the energy transferred to SPPs becomes trapped within the OLED and dissipates as heat, negatively affecting light extraction efficiency. [Mikami, A *et al.*, Optics and Photonics journal, 06, 226-232 (2016) and Park, H-u *et al.*, Organic Electronics, 105 (2022)]

We calculated the dissipated power spectra for a bottom emission normal OLED with thick Al cathode and the transparent OLED structure used in this study. The material and structure of organic layers and anode are the same, and only the cathode is different. The simulation results revealed that the outcoupling radiation is limited by the wavevector of the radiation. Radiation with a wavevector of $u < 0.56$ can escape to the air, while radiation with $0.56 < u < 1$ cannot escape due to the substrate mode and waveguide mode. Furthermore,

radiation with u > 1 is absorbed by SPP in the metal and cannot escape from the device; the evanescent peaks in the yellow zone belong to the SPP.

As shown in the curves, a single SPP 1 peak occurs in the normal OLED, due to the Fabry-Pérot (FP) cavity at the organic/Al interface. In contrast, in the transparent OLED, both the FP cavity SPP 1 peak between the organic material and metal and a hybrid SPP 2 peak arising from the multi-layer structure including organic, metal, and air are observed. A significant difference is observed at the SPP 2 peak, which is due to the increased energy loss from surface plasmons. As a result, the transparent OLED exhibits lower efficiency compared to the normal OLED. [Zang, C. *et al.*, Light Science and Application, 10, 116 (2021)] The asymmetric luminance between BE and TE in the transparent OLED is due to the difference in SPP 1 peak.

These results are attributed to the modification in the optical cavity depending on the cathodes. In the normal OLED structure, commercial ITO and high-reflectivity Al electrodes are used, which minimizes interference and power loss when light is emitted towards the ITO. However, in the transparent OLED, both reflection and transmission occur at the transparent cathode, forming an optical cavity. This results in multiple beam and wide-angle interference, leading to lower efficiency.

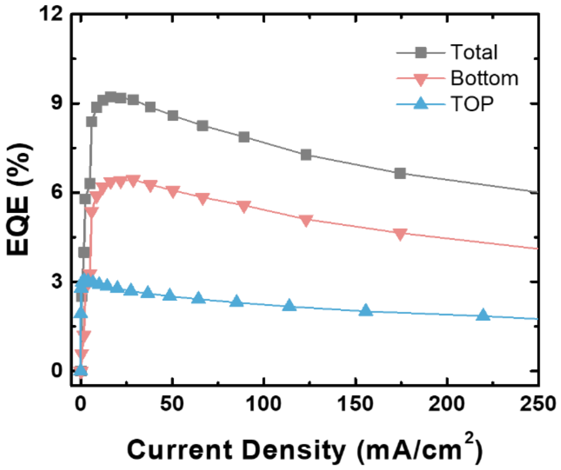

Supplementary Figure S9. External quantum efficiency (EQE) as a function of current density for the bidirectional viewing device. The cavity structure may not be optimized in this device, as indicated in the simulation result in Fig. S7.

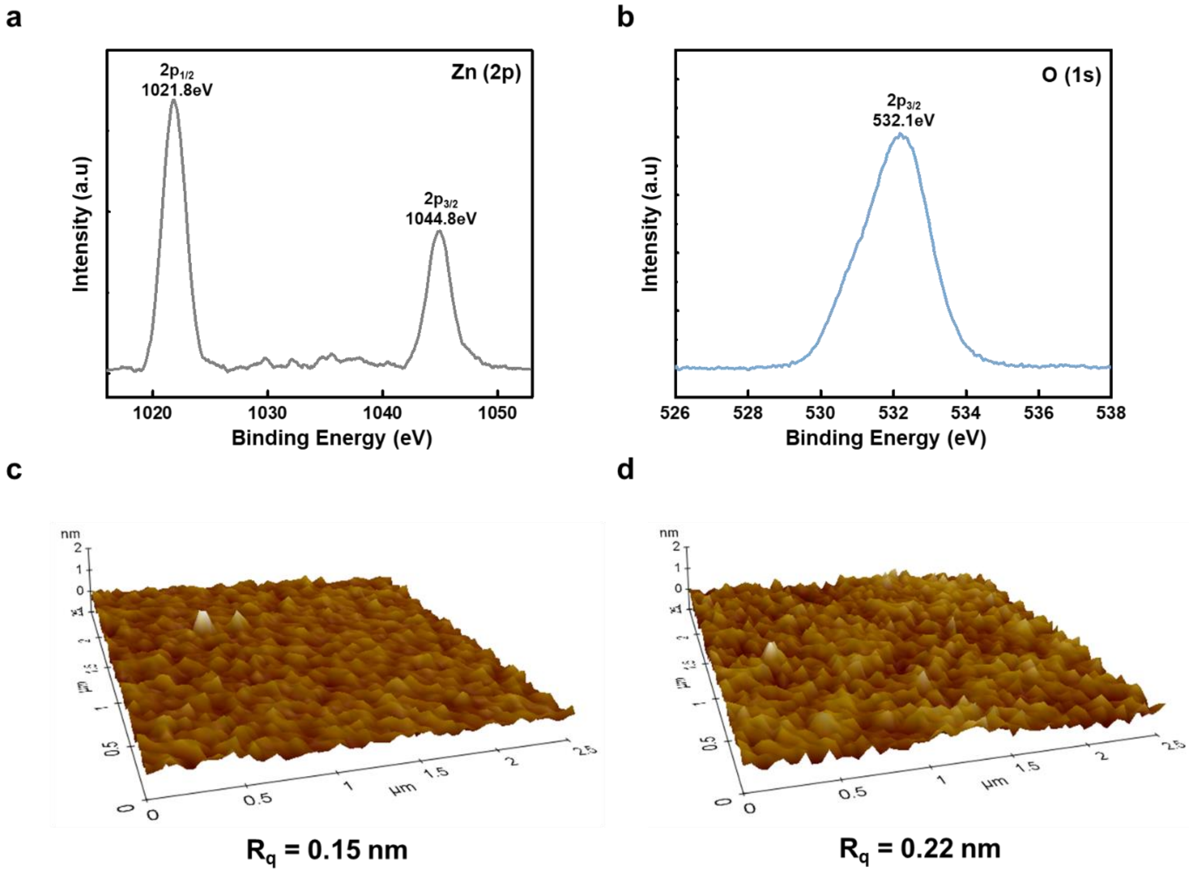

Supplementary Figure S10 a-b. Zn 2p and O 1s XPS spectra of ZnO thin film, respectively. **c-d.** Atomic force microscopic (AFM) images and surface roughness of Bphen:Liq (20 nm) (c), and Bphen:Liq (20 nm) /ZnO (3 nm) (d).

The surface roughness increasement was approximately 0.07 nm after the deposition of 3 nm ZnO on Bphen:Liq layer, which is negligible. The surface of ZnO on Bphen:Liq was very smooth with the roughness of 0.22 nm, which can help the formation of uniform thin metal layer on it.

Supplementary Table S1. Figure of merit (FOM) of Yb:Ag electrodes with varying Yb ratio at 13 nm thickness

Yb Doping concentration	T_{toal} [%]	T_{510nm} [%]	Sheet resistance [Ω/□]	FOM [$x10^{-3}Ω^{-1}$]
10%	77.26	80.18	18.84	5.82
8%	81.17	84.48	17.55	10.55
6%	80.80	83.73	13.78	12.29
4%	77.85	83.73	15.25	11.10
0%	75.3	80.34	26.02	4.30

Supplementary Table S2. Performance of various metal based transparent cathode

Cathode	Thickness (nm)	Transmittance (%)		R□ (Ω/□)	R_q(nm)	ϕ (eV)	Ref
ZnO/Yb:Ag	15	73.0[a]	(86.8)[b]	11.61	0.53	3.86	This work
Ni:Ag	7	79.3[a]	-	18.92	0.57	4.5	15
Cu:Ag	7	80.7[a]	-	12.33	0.42	-	15
Mg:Ag	20	49.7[a]	(48.6)[b]	5.2	-	4.7	16
Ca:Ag	18	64[a]	-	21	1.6	-	17
Al/Al:Ag	14	86.8[a]	(83.5)[b]	7	1.16	-	35

a. Average transmittance at the visible wavelength range

b. Transmittance at the characteristic wavelength in each work.

Supplementary Movie S1. Example of driving a display in the one-side-view mode, identicaldual-view mode, and heterogeneous dual-view mode.
